# The General Practice Assessment Questionnaire (GPAQ) – Development and psychometric characteristics

**DOI:** 10.1186/1471-2296-9-13

**Published:** 2008-02-20

**Authors:** Nicola Mead, Peter Bower, Martin Roland

**Affiliations:** 1 National Primary Care Research and Development Centre, University of Manchester, Williamson Building, Manchester M13 9PL, UK

## Abstract

**Background:**

Continual quality improvement in primary care is an international priority. In the United Kingdom, the major initiative for improving quality of care is the Quality and Outcomes Framework (QoF) of the 2004 GP contract. Although the primary focus of the QoF is on clinical care, it is acknowledged that a comprehensive assessment of quality also requires valid and reliable measurement of the patient perspective, so financial incentives are included in the contract for general practices to survey patients' views. One questionnaire specified for use in the QoF is the General Practice Assessment Questionnaire (GPAQ). This paper describes the development of the GPAQ (with post-consultation and postal versions) and presents a preliminary examination of the psychometric properties of the questionnaire.

**Methods:**

Description of scale development and preliminary analysis of psychometric characteristics (internal reliability, factor structure), based on a large dataset of routinely collected GPAQ surveys (n = 190,038 responses to the consultation version of GPAQ and 20,309 responses to the postal version) from practices in the United Kingdom during the 2005–6 contract year.

**Results:**

Respondents tend to report generally favourable ratings. Responses were particularly skewed on the GP communication scale, though no more so than for other questionnaires in current use in the UK for which data were available. Factor analysis identified 2 factors that clearly relate to core concepts in primary care quality ('access' and 'interpersonal care') that were common to both version of the GPAQ. The other factors related to 'enablement' in the post-consultation version and 'nursing care' in the postal version.

**Conclusion:**

This preliminary evaluation indicates that the scales of the GPAQ are internally reliable and that the items demonstrate an interpretable factor structure. Issues concerning the distributions of GPAQ responses are discussed. Potential further developments of the item content for the GPAQ are also outlined.

## Background

Continual quality improvement in primary care is an international priority. In the United Kingdom, there have been major initiatives to improve the quality of clinical and organisational aspects of care, most recently through implementation of the Quality and Outcomes Framework (QoF) of the 2004 General Practitioner (GP) contract [1].

Although a large proportion of quality improvement efforts are appropriately targeted at indicators of clinical quality, comprehensive assessment additionally requires taking account of the patient perspective [2,3]. Valid and reliable measurement of patients' perceptions of the quality of their care is therefore of fundamental importance [4].

A number of relevant questionnaires are currently available for assessing patients' views [5-8], but only two are currently specified for use in the GP contract: the Improving Practice Questionnaire [9] and the General Practice Assessment Questionnaire [10].

The IPQ is a short (two sides of A4) questionnaire which includes 27 items dealing with access to the practice; continuity of care; interpersonal communication; care provided by reception staff; and services provided by the practice.

The GPAQ is the result of a relatively long development process. The first version of the questionnaire, called the General Practice Assessment Survey (GPAS), was developed in 1997 as a valid, easy to use measure of patients' perceptions of the following critical aspects of general practice: availability and accessibility of care; technical and interpersonal competence (both of GPs and practice nurses); coordination and continuity of care [11].

The GPAS was itself based on a parent questionnaire developed in the United States called the Primary Care Assessment Survey (PCAS). At that time, PCAS was the most well-validated primary care assessment tool in the world. PCAS has excellent psychometric properties and is sensitive to the care received by different population groups [12], to the quality of care in different types of health care systems [13,14], to different types of doctors [15], to outcomes such as adherence, satisfaction and health status [16] and predicts voluntary disenrollment from primary care practices [17].

The GPAS has a more limited evidence base. It has been found to be internally consistent and reliable over time [11] and has an interpretable and replicable factor structure [18]. Scores are sensitive to patient demographic characteristics such as ethnicity and age [19,20] and to characteristics of the practice [21-23]. GPAS subscales correlate well with self-reported 'overall satisfaction' and 'enablement' scores [18] and are also related to objective outcomes in diabetes [24].

The GPAQ is currently used by thousands of general practices in the United Kingdom as part of the QoF. Given the changes that occurred during the evolution from GPAS to GPAQ, there have been calls to re-assess the reliability and validity of the new GPAQ questionnaire [25]. This paper presents:

• an outline of the development process that led to the GPAQ

• preliminary data concerning its psychometric characteristics

## Methods

### Description of the development of the GPAQ

The GPAQ was developed from its parent GPAS questionnaire in response to research findings and developing policy in the United Kingdom. In 2002, the GPAS was recommended as a tool for use as part of the 'Patient Experience' domain of the QoF, which incentivises practices to survey patients about their care. However at seven sides of A4, the GPAS was acknowledged as being too long to ensure optimal response among users.

Thus, as a first step in developing the GPAQ from the GPAS, statistical analyses of a dataset of over 20,000 GPAS responses identified those items that were poorly discriminating or potentially redundant due to high inter-item correlations. These were subsequently incorporated into a short survey along with 13 other items of general practice care that might potentially be included in the new GPAQ. The survey was sent to the clinical governance leads of 100 randomly selected English Primary Care Trusts (PCTs) who were asked to select the four items they felt it was most important to include in the GPAQ. Fifty-three PCTs responded. Table 1 shows the percentage frequency with which each aspect of care was endorsed. In addition, respondents gave additional free-text suggestions for what they would like to see in the GPAQ.

**Table 1:** Results of 2003 survey of PCT clinical governance leads (n = 53)

**Item of general practice care for potential inclusion in GPAQ**	**Percentage of respondents identifying item as one of four highest priorities**
1. Ability to get an urgent (same day) appointment with a GP	60
2. Ability to see a GP of the patient's own gender	13
3. Ability to see a GP of the patient's own ethnic group	0
4. Ability to see a GP of the patient's choice	38
5. Ability to get a home visit, if needed, when the practice is open	23
6. Ability to get a home visit, if needed, when the practice is closed	23
7. Cleanliness/comfort of waiting room/consulting rooms/patient facilities, etc.	19
8. Availability of areas in the practice where the patient can discuss things privately with staff	36
9. The degree to which the patient feels involved in decision making about his/her medical care	68
10. The GP's knowledge of the patient's medical history	21
11. The patient's trust in the GP	25
12. The degree to which confidential information about the patient is respected and protected by practice staff	32
13. The degree to which the patient is kept informed of the results of any tests and investigations	36
14. Ability to get referral to a specialist when the patient feels it is necessary	19
15. The degree to which the GP prepares the patient for what to expect from specialist referral/hospital care	25
16. Cooperation/communication between the GP and other NHS staff involved in the patient's care	40

As a result of the survey and wider discussions among the academic team, new evaluative items were incorporated into the 'communication skills' scale of the GPAQ concerning how well the GP puts the patient at ease during physical examinations (tapping into issues raised by survey respondents relating to respect, trust and privacy), and the degree to which the GP involves the patient in decision-making (rated important by 68% of respondents). Furthermore, an existing GPAS item on availability of urgent (same day) appointments was retained in the GPAQ. Sixty per cent of PCT respondents rated it important, even though it was poorly discriminating as over 85% of patients answered 'yes'.

In a further development, to make use of the GPAQ as flexible as possible, two different versions were created. The 'consultation version' is designed to be completed by patients aged 16 or over after they have seen a GP. It contains 25 items evaluating key domains of general practice, including access, continuity of care, the GP's communication skills and the patient's post-consultation enablement (i.e. the degree to which they feel more able to understand and cope with their health problem and keep themselves healthy). These three enablement items were derived from the six-item Patient Enablement Instrument [26,27] and were included as a measure of consultation *outcome *from the patient's perspective. In addition, there are 7 items collecting a range of health and socio-demographic information from respondents, and a free text section for patient comments.

The 33-item postal version is designed to be sent out to a random sample of adult patients from the practice list for completion at home. It contains many of the same items as the consultation version (i.e. those relating to access, practice receptionists, continuity of care and patient health and socio-demographic characteristics). In this version however, patients are not asked to rate the communication skills of a named GP within a specific consultation; rather, patients respond in a general context about their *usual doctor *(or the one they know best from the practice). The postal version also substitutes enablement questions with items about the quality of nursing care at the practice.

An advantage of the 'consultation version' is that it can be used to derive individual general practitioner scores (e.g. for reaccreditation purposes). However, surveying consulting patients assesses a different population than if the 'postal version' is mailed to a random sample of patients.

Finally, development of the GPAQ involved some minor changes to question wording and response categories. For example, the response options in items about appointment waiting times were altered to provide greater accuracy and reflect the government's new 48-hour target [28] – so '2–3 days' became 'within 2 working days' and '4–5 days' was split into 'within 4 working days' and '5 or more working days'.

Analyses of the GPAQ version 1.0 data collected from United Kingdom practices during the first year of the new GP contract (2004–5) lead to the subsequent removal in version 2.0 of the 'Overall satisfaction' item. This was partly due to evidence that a significant minority of patients had misinterpreted the response categories to this item (which was scored in reverse to other items in the GPAQ 1.0). However, it was also felt by the research team that inclusion of a global rating of 'Overall satisfaction' was not in line with the ethos of the GPAQ as a tool for quality *improvement*, since the item provides a practice with little useful information on which to act, unlike the other more specific items in the questionnaire.

### Dataset

As noted earlier, the GP contract in the United Kingdom introduced a financial incentive for practices to survey patients using either the GPAQ or IPQ. GPAQ data from practices across the United Kingdom have been collected centrally by the National Primary Care Research and Development Centre at the University of Manchester and form the basis of the current analysis. Specifically, the dataset comprises 190,038 individual patient responses to the post consultation version of the GPAQ collected across 1,031 United Kingdom general practices during the 2005–6 contract year, plus 20,309 patient responses to the postal version from 149 practices. In some cases, practices have administered the postal version of the GPAQ to patients attending the surgery rather than mailing out them to a random sample, and these data have been ignored for the present analysis. The mean number of patients surveyed per practice was 184 (range 1 to 1088) for the post consultation version, and 136 (range 10 to 505) for the postal version.

### Analysis

The data used for the analysis were collected for routine quality improvement purposes, rather than for a specific research project. Given the potential lack of rigour and control around such routine data collection, concerns about potential bias exist. Therefore, an initial analysis compared characteristics of the respondents in the GPAQ sample to characteristics of patients in the General Household survey (a continuous survey carried out by the Office for National Statistics which collects information on a range of topics from people living in private households in Great Britain) [29] and the Census [30].

Scores for the GPAQ scales and individual items were computed in line with published procedures, where zero is the lowest possible score and 100 is the highest possible score (i.e. scores are interpreted as a percentage of the maximum possible score). Note that some items in both versions of GPAQ are included purely for descriptive purposes and do not contribute to the scale scores.

The dimensional structure of the GPAQ was assessed using factor analysis, in which the observed covariance among multiple variables are described in terms of a smaller number of hypothetical 'factors'. A number of methods are available for factor analysis, which differ in terms of their goals and statistical features. One of the most common techniques is principal axis factor analysis. This technique uses the factor model whereby common variance is analysed and variance due to unique and error variance removed [31].

The analysis followed recommended procedures [32]. First, a principal components analysis was conducted, and the scree plot from this preliminary analysis was examined to identify the number of factors. The full principal axis factor analysis was then conducted with this number of factors. The solution was rotated using the varimax rotation procedure to produce 'orthogonal' (i.e. uncorrelated) factors. Rotation is designed to improve the interpretability of factors by finding the solution which maximises the number of factors with a few high loadings, and the rest close to zero (so called 'simple structure'). This procedure was the same as that used in published factor analyses of the original GPAS [18].

The larger data set available for the post consultation version of the questionnaire was used initially. The factor analysis was then repeated on the postal version, using identical analytic procedures.

The reliability of the GPAQ was evaluated using Cronbach's alpha for multi-item scales. Cronbach's alpha is a measure of the internal consistency of a scale, based on the average inter-item correlation.

The interpretability and utility of the GPAQ was evaluated through an examination of the distribution of responses across response categories.

All data manipulation and analysis was conducted using the Statistical Package for the Social Sciences (SPSS version 14.0).

## Results

Table 2 shows the demographic characteristics of the respondents to each version of the questionnaire. Comparison of the demographic characteristics in Table 2 with data available from the General Household Survey (not shown) indicate that females are somewhat overrepresented among respondents (1.8 females to every male respondent, compared with a female to male ratio of 1.6 to 1 reported in the GHS). Proportionately fewer female GPAQ respondents are economically inactive compared with the General Household Survey sample, particularly in the 45–60 age group. However, male GPAQ respondents are broadly similar to in terms of age and employment status to males who reported consulting a GP in the General Household Survey. The larger post consultation GPAQ sample does appear to be representative of the total United Kingdom population in terms of ethnicity when compared to census rates (Census vs. GPAQ post consultation: White 92.1 vs. 92.2%; Asian or Asian British 4.0 vs. 3.7%; Black or Black British 2.0 vs. 1.8%; Chinese 0.4 vs. 0.3%; Mixed 1.2 vs. 1.1%; Other ethnic group 0.4 vs. 0.9%). The patients included in the postal sample are less representative in terms of ethnicity.

**Table 2:** Characteristics of patients in the analytic sample

**Socio-demographic characteristics**	**Consultation GPAQ**	**Postal GPAQ**
Total *n*	190,038	20,309
No. of practices	1,031	149
Mean age in years	50.3	54.2

GP consultations in the past year:		
- % 'None'	4.2	8.0
- % 'Once or twice'	22.5	28.7
- % 'Three or four times'	30.4	29.6
- % 'Five or six times'	21.0	18.2
- % 'Seven times or more'	21.9	15.4

% female	64.7	61.4
% with a long-term illness, disability or infirmity	51.0	48.2

Ethnicity:		
- % white	92.2	86.8
- % Asian/Asian British	3.7	5.9
- % Black/Black British	1.8	5.0
- % Mixed	1.1	1.3
- % Chinese	0.3	0.1
- % Other ethnic group	0.9	0.7

Employment:		
- % employed	48.4	35.3
- % unemployed	2.5	1.8
- % in full-time education	3.4	2.3
- % unable to work due to long-term sickness	7.2	5.2
- % looking after home/family	9.6	7.1
- % retired	27.5	45.0
- % other	1.6	3.3

% living in rented accommodation	28.9	25.4

Note: missing values were excluded from the denominator when calculating percentages for each category

In the analysis of the post consultation GPAQ, analysis of the scree plot identified 3 factors. The three factors accounted for 66.1% of the variance. The rotated factor matrix is shown in Table 3. Items loading >0.3 on a factor are considered substantive and are bolded in the Table. Similarly, the scree plot also identified 3 factors in the analysis of the postal GPAQ. These factors accounted for 73.0% of the variance. The factor loadings are shown in Table 4.

**Table 3:** Rotated factor matrix of the analysis of the post consultation version of the GPAQ

**Item**	**Component 1**	**Component 2**	**Component 3**
Receptionists	.231	**.522**	-.061
Availability of specific GP	.071	**.840**	-.052
Availability of any GP	.156	**.755**	-.025
Waiting times at surgery	.210	**.625**	-.072
Continuity of care	.295	**.593**	-.052
GP questioning	**.836**	.236	-.080
GP attention	**.878**	.214	-.098
GP putting you at ease	**.734**	.156	.020
GP involving you in decisions	**.786**	.182	.012
GP explanations	**.845**	.188	-.042
GP spending time with you	**.815**	.241	-.113
GP patience	**.869**	.190	-.061
GP caring and concern	**.863**	.204	-.101
Able to understand your problem	-.077	-.061	**.822**
Able to cope with your problem	-.065	-.066	**.927**
Able to keep yourself healthy	-.044	-.077	**.781**

GPAQ includes a mix of report and evaluation items. Only evaluation items are included in the scoring of GPAQ and were used for the factor analysis. Cases with missing data on any of the included variables were excluded from the analysis (analysis included 119467 from 190038). It should be noted that some data is not completed (and are therefore 'missing') because the items are not appropriate to the individual patient.

**Table 4:** Rotated factor matrix of the analysis of the postal version of the GPAQ

**Item**	**Component 1**	**Component 2**	**Component 3**
Receptionists	.256	**.550**	.260
Availability of specific GP	.110	**.861**	.111
Availability of any GP	.199	**.737**	.166
Waiting times at surgery	.224	**.658**	.172
Continuity of care	**.352**	**.598**	.160
GP questioning	**.830**	.242	.206
GP attention	**.869**	.219	.189
GP putting you at ease	**.781**	.198	.179
GP involving you in decisions	**.773**	.202	.176
GP explanations	**.851**	.212	.204
GP spending time with you	**.798**	.261	.192
GP patience	**.863**	.213	.175
GP caring and concern	**.863**	.230	.174
How well nurse listens	.246	.244	**.869**
Quality of care from nurse	.255	.230	**.898**
How well nurse explains health problems	.243	.234	**.867**

GPAQ includes a mix of report and evaluation items. Only evaluation items are included in the scoring of GPAQ and were used for the factor analysis. Cases with missing data on any of the included variables were excluded from the analysis (analysis included 9,807 from 20,309). It should be noted that some data is not completed (and are therefore 'missing') because the items are not appropriate to the individual patient.

**Figure 1:**
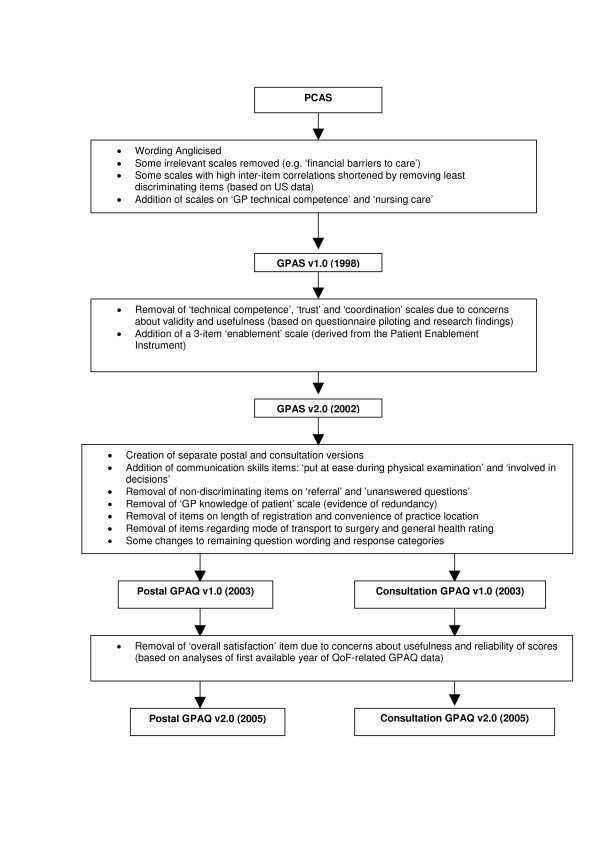
Development of GPAQ Version 2.0.

Estimates of internal reliability were uniformly high (0.88 to 0.97 in the postal version, 0.86 to 0.97 in the consultation version). The development of the GPAQ (Figure 1) involved the addition of two communication items on shared decision making and the ability of the doctor to put the patient at ease during the physical examination. Analysis showed that the reliability of the overall communication scale was not significantly changed if either of these items were deleted.

**Figure 2:**
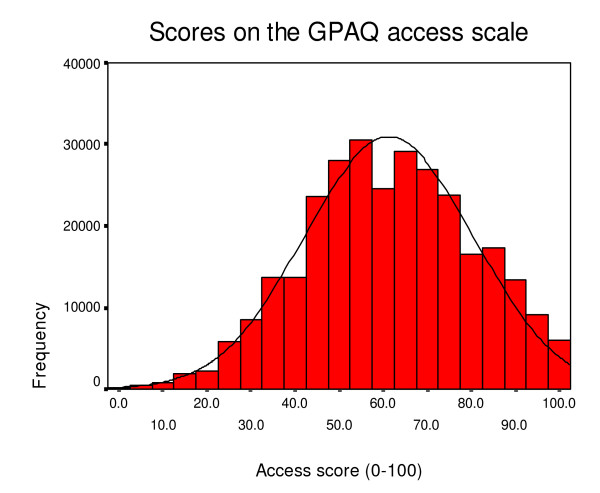
Distribution of access scores.

**Figure 3:**
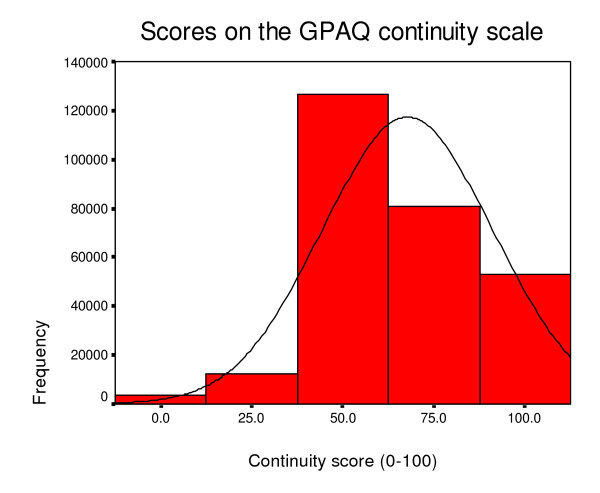
Distribution of continuity scores.

**Figure 4:**
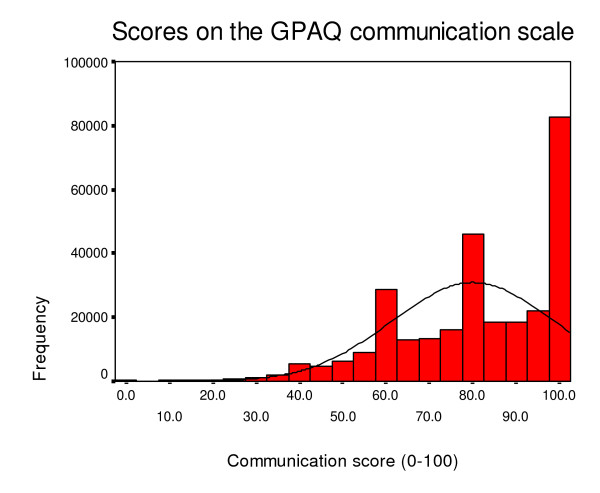
Distribution of communication scores.

Table 5 and 6 show descriptive data and score distributions for the two versions of the GPAQ questionnaire. As with the GPAS, respondents tend to report generally favourable ratings (see Figures 2, 3 and 4 which show the distribution of scores on the access, continuity and communication scales of the post consultation version). The distribution is especially skewed with respect to the communication scale. The skew statistic was -0.115, -0.450 and -0.999 respectively on the access, continuity and communication scales of the post consultation version. Apart from responses to the access scale, between one fifth and one third of respondents score at the maximum.

**Table 5:** Descriptive data and score distributions for the post consultation GPAQ version 2 (n = 190,038)

**GPAQ scale/item**	***N***	**Minimum**	**Maximum**	**Mean SD**	**% respondents with lowest possible score**	**% respondents with highest possible score**	**Alpha**
Access (6 items)	186,684	0	100	62.3 (18.6)	0 (n = 75)	2.0 (n = 3668)	0.86 (n = 82,519*)
Receptionists (1 item)	188,613	0	100	77.2 (20.2)	0.5 (n = 947)	30.7 (n = 57,842)	n/a
Continuity of care (1 item)	171,465	0	100	68.8 (23.0)	1.1 (n = 1821)	20.1 (n = 34,481)	n/a
Communication (8 items)	182,777	0	100	82.5 (17.5)	0.2 (n = 322)	28.3 (n = 51,714)	0.97 (n = 142,934)
Enablement (3 items)	144,740	0	100	65.5 (34.8)	11.8 (n = 17,072)	39.0 (n = 56,516)	0.91 (n = 124,974)

* note that sample size for reliability analysis is much reduced due to the fact that 'don't' know/never needed to' responses to the two GPAQ telephone access questions are treated as missing for scale scoring purposes.

**Table 6:** Descriptive data and score distributions for the postal GPAQ version 2 (n = 20,309)

**GPAQ scale/item**	***N***	**Minimum**	**Maximum**	**Mean SD**	**% with lowest possible score**	**% with highest possible score**	**Alpha**
Access (6 items)	19,418	0	100	58.8 (19.5)	0 (n = 11)	1.8 (n = 359)	0.88 (n = 8,277)*
Receptionists (1 item)	20,024	0	100	72.6 (21.8)	0.9 (n = 184)	23.7 (n = 4746)	n/a
Continuity of care (1 item)	18,290	0	100	66.6 (23.5)	1.6 (n = 288)	17.7 (n = 3240)	n/a
Communication (8 items)	18,463	0	100	77.0 (19.5)	0.1 (n = 24)	19.2 (n = 3554)	0.97 (n = 16,106)
Nursing care (3 items)	13941	0	100	76.4 (18.0)	0.2 (n = 28)	21.5 (n = 3003)	0.96 (n = 13,777)

* note that sample size for reliability analysis is much reduced due to the fact that 'don't' know/never needed to' responses to the two GPAQ telephone access questions are treated as missing for scale scoring purposes.

## Discussion

As noted earlier, the data used for the analysis were collected for routine quality improvement purposes, and concerns about potential bias exist. Little data was available on the procedures used to survey patients in individual practices, and it is possible that practices were selective in deciding which patients to ask to complete a survey. Therefore, it would be useful to attempt to replicate the findings with representative samples recruited for research. However, it should also be noted that there is evidence that GPs are not good at predicting patients' likely response to such questionnaires, which may mean that even if the surveys are selective, they may still adequately sample a range of opinion [33]. Finally, GPAQ data from patients are clustered within doctors and within practices, and in the future more sophisticated analyses may be required to take account of this clustering in the analysis of the factor structure of the scale.

Generally, the data suggest that the GPAQ questionnaire, like the parent GPAS, is acceptable, reliable and has an interpretable factor structure. There are some potential problems with the distribution of scores, especially in relation to the communication scale. However, these problems are common in questionnaires assessing patients' views of medical care, and it is not clear whether they represent some sort of response set [34], or reflect genuine high levels of satisfaction. For the GPAQ communication scales, a fifth to a third of patients scored at the maximum. This suggests that the questionnaire is at least as discriminating as other questionnaires in current use in the United Kingdom. For example, of respondents to the Healthcare Commission/Picker Europe primary care survey in 2005 [35], 82% said that their doctor listened carefully to them, 74% said that their doctor definitely spent enough time with them, and 76% said that their treatment was explained in a way that they could understand. In each case, these represent the most positive response available to patients in the survey. For the IPQ questionnaire, eleven out of twelve communication items have average scores between 'very good' and 'excellent' on a five point scale [36].

Responses to the additional items added to the GPAQ questionnaire are consistent with the other questionnaire items concerning communication. Indeed, the associations are high enough that it might be questioned whether additional information is gained by the addition of these new items. Partly, their addition reflects pragmatic criteria, as practices can use the responses to individual items to reflect on their performance and consider change, and a greater range of item content may be of greater utility, even if the items are highly correlated at the level of the overall sample.

The factor analyses of both versions of the GPAQ showed a similar, interpretable structure, with separate factors relating to 'access' and 'communication'. The third factors represented 'enablement' in the post consultation version and 'nursing care' in the postal version. These results were in line with previous factor analyses of the GPAS [18]. The item relating to the perceived quality of reception staff related to the 'access' factor. In both analyses, only the continuity item shows a problematic pattern of loadings, as it loaded heavily on access, but also had high loadings (0.295 and 0.359) on 'communication'. Although such a pattern is generally seen as problematic in factor analysis, it should be noted that conceptually it makes some sense, as the ability to get an appointment with the same doctor might be viewed as an access issue, whereas the relational continuity that such access allows would theoretically relate to doctor-patient communication.

Questionnaire development and validation is an ongoing process, and there are a number of further developments of the GPAQ which are required.

The small scale survey of primary care management staff used in the development of the GPAQ found that managers were also very interested in the addition of items related to 'co-operation/communication between the GP and other NHS staff involved in patient care' and 'the degree to which the patient is kept informed of the results of tests/investigations'. Both these items refer to the issue of co-ordination of care. There is currently much interest in that issue, because changes in the delivery of care associated with the new GP contract (such as increasing specialisation and vertical integration in the management of chronic disease) have tended to fragment care over multiple professionals and services, which may threaten the patient experience of continuity and co-ordination [37]. It is important that questionnaires such as the GPAQ can keep pace with such developments, and the addition of items dealing with the issue of co-ordination are a priority. However, developing such a measure from the patient's perspective is likely to be a significant challenge.

Similarly, increasing emphasis is being placed on the role of self-care in chronic disease management [38,39]. However, primary care staff vary in their ability to provide such self-care advice because of a variety of professional and contextual barriers [40]. Although some of the skills underlying self-care support may relate to those concerning interpersonal care more generally, there are likely to be important differences, so future versions of the GPAQ might usefully include patient assessments of the effectiveness of GP self-care support.

Although the GPAQ has a developing body of data concerning validity, there are some areas that would benefit from further work. This is especially true of the validation of the GPAQ against external criteria [25]. Data concerning the ability of GPAQ to predict patient behaviours are limited, and there is no equivalent of the data showing that PCAS scores predict voluntary disenrollment, partly because the current organisation of primary care services in the United Kingdom does not encourage such obvious displays of patient dissatisfaction. Although a number of external criteria were discussed in the introduction, identification of definitive external criteria that provide an unambiguous test of patient assessment scales such as GPAQ is problematic [41].

Responsiveness relates to the ability of an instrument to detect meaningful change in the construct it is supposed to measure. Such a characteristic is of importance if the GPAQ is to be used as a measure of the effects of interventions. For example, trials have been conducted of the use of feedback of patient questionnaire scores as a way of improving quality of care from the patient perspective [42,43], which require that questionnaires such as the GPAQ are sensitive to changes in the delivery of care.

At present, the existence of large, representative datasets of scores provides an approach to interpretability based on *benchmarking *i.e. comparing scores of individual practices with data from the national samples. According to the GPAQ manual, when reporting data, scores should be noted when they are 10 points above and below the mean values on each scale. The 10 point difference is relatively arbitrary. Other approaches might be better, such as reporting the 10th and 90th centiles. Another approach would base the difference on a measure such as the standard error of measurement. However, this requires estimates of reliability. Most of the data on reliability of the GPAQ relates to measures of internal consistency, and the use of these estimates as measures of reliability is not recommended as they do not take account of important forms of variation [44]. Only one published estimate of test-retest reliability is available for the GPAS, based on small sample sizes [11]. Using those values would suggest standard errors of measurement of 8.6 for access and 7.7 for communication. It is still not clear whether these thresholds are meaningful. This relates to the more general problem of *calibration*. The GPAQ scores (like many health services research questionnaires) are not expressed in a metric that is intuitively meaningful. Differences between practices of 10 points, or a standard deviation, are not easy to conceptualise in terms of patient experience. For example, what is communication like for a patient in a practice 10 points below the national benchmark? Further work on the interpretability of the GPAQ might increase its utility for clinicians, managers, policy makers and patients.

## Conclusion

Initial analyses suggest that the new GPAQ scale meets basic psychometric criteria. A more comprehensive assessment of the psychometric quality of the scale will require additional tests. It is likely that future versions of the GPAQ will require the addition of new items and scales in order to ensure that the GPAQ is relevant to the changing policy context and service delivery in the National Health Service.

## Competing interests

The GPAQ is free to use for NHS staff, but commercial companies selling patient evaluation services are required to pay a license fee. Funds derived from selling the GPAQ in these instances are received by the National Primary Care Research and Development Centre and used to fund support for the GPAQ and related research. No individual gains directly from the use of the GPAQ.

## Authors' contributions

MR, NM and PB were all involved in the development of the GPAS and GPAQ questionnaires. NM managed the database and conducted the bulk of the analysis. PB conducted the factor analysis. NM and PB wrote the paper, which was revised by MR. All authors read and approved the final manuscript

## Pre-publication history

The pre-publication history for this paper can be accessed here:


